# Quantitative estimation of DNA damage by photon irradiation based on the microdosimetric-kinetic model

**DOI:** 10.1093/jrr/rrt222

**Published:** 2014-02-09

**Authors:** Yusuke Matsuya, Yosuke Ohtsubo, Kaori Tsutsumi, Kohei Sasaki, Rie Yamazaki, Hiroyuki Date

**Affiliations:** 1 Graduate School of Health Sciences, Hokkaido University, Kita-12, Nishi-5, Kita-ku, Sapporo 060-0812, Japan; 2 Hokkaido PWFAC Sapporo-Kosei General Hospital, Kita-3 Higashi-8, Chuo-ku, Sapporo 060-0033, Japan; 3 Faculty of Health Sciences, Hokkaido University, Kita-12, Nishi-5, Kita-ku, Sapporo 060-0812, Japan; 4 Graduate School of Engineering, Kyoto University, Kyoto Daigaku-Katsura, Nishikyo-ku, Kyoto 615-8530, Japan

**Keywords:** microdosimetric-kinetic model, γ-H2AX foci, double-strand breaks, RBE_37_

## Abstract

The microdosimetric-kinetic (MK) model is one of the models that can describe the fraction of cells surviving after exposure to ionizing radiation. In the MK model, there are specific parameters, *k* and *y*_*D*_, where *k* is an inherent parameter to represent the number of potentially lethal lesions (PLLs) and *y*_*D*_ indicates the dose-mean lineal energy in keV/μm. Assuming the PLLs to be DNA double-strand breaks (DSBs), the rate equations are derived for evaluating the DSB number in the cell nucleus. In this study, we estimated the ratio of DSBs for two types of photon irradiation (6 MV and 200 kVp X-rays) in Chinese hamster ovary (CHO-K1) cells and human non-small cell lung cancer (H1299) cells by observing the surviving fraction. The estimated ratio was then compared with the ratio of γ-H2AX foci using immunofluorescent staining. For making a comparison of the number of DSBs among a variety of radiation energy cases, we next utilized the survival data in the literature for both cells exposed to other photon types, such as ^60^Co γ-rays, ^137^Cs γ-rays and 100 kVp X-rays. The ratio of DSBs based on the MK model with conventional data was consistent with the ratio of γ-H2AX foci numbers, confirming that the γ-H2AX focus is indicative of DSBs. It was also shown that the larger *y*_*D*_ is, the larger the DSB number is. These results suggest that *k* and *y*_*D*_ represent the characteristics of the surviving fraction and the biological effects for photon irradiation.

## INTRODUCTION

Photon beams are in widespread use in radiotherapy for eradicating cancers. The biological effect of the photon beams is currently standardized as the relative biological effectiveness (RBE). The RBE has been conventionally regarded as unity in any energy range, but it is also known that the effects of radiation depend on the absorbed dose [Gy], radiation type and dose rate [1–3]. As for the cellular-level response, we are able to evaluate the effects of radiation via cell surviving fraction using the method of colony formation assay reported by several investigators [4, 5]. The primary target for causing cell damage by radiation is the cell nucleus, particularly the DNA [6, 7]. DNA lesions by irradiation may lead to cell death. These lesions are classified into two types: single-strand breaks (SSBs), and double-strand breaks (DSBs). It is known that most SSBs are repairable in a relatively short period of time and do not lead to cell death directly, while DSBs promote lethal damage effectively.

For describing cell survival after irradiation, several models and formulae have been reported [8–11]. Among them, the linear–quadratic (LQ) model is currently the most widely used in radiotherapy. In the LQ model, the surviving fraction is given by *S =* exp(−*αD* − *βD*^*2*^), where *α* and *β* are the proportionality factors to the absorbed dose (*D*) [Gy^−1^] and the dose squared (*D*^2^) [Gy^−2^], respectively. In many cases, the LQ curve fits fairly well to the survival data. However, the implications involved in the terms are not necessarily trivial. The microdosimetric-kinetic (MK) model (presented by R.B. Hawkins) can evaluate the surviving fraction in terms of microdosimetry [12–14]. This model takes into account the spatial distribution of the energy deposition of radiation [which includes the dose mean lineal energy, *y*_*D*_ (in keV/μm)] for expressing the difference of radiation energy [15]. The *y*_*D*_ value can be measured using the tissue-equivalent proportional counter (TEPC) and calculated by Monte Carlo simulation [16–18]. Another advantage of the MK model is that it contains the inherent parameter (*k*) that implies the number of potentially lethal lesions (PLLs) per cell nucleus per Gy. *k* is defined as the characteristic parameter that depends on radiation energy [19]. These parameters (*k*, *y*_*D*_) provide an explanation of the curving nature in the LQ model and an estimation of damage degree, e.g. the number of DSBs.

From an experimental point of view, we can observe the DSBs visually with immunofluorescent staining using the γ-H2AX antibody, 53BP1 or MDC1 [20, 21]. There have been several investigations concerning the γ-H2AX foci, and the number of γ-H2AX foci has been observed with time-lapse images [22]. Beyreuther *et al*. suggest that the number of γ-H2AX foci peaks at 30 min after irradiation, and the number of DSBs is larger for lower photon beam energy [23]. As described above, we have quantified the number of foci from a theoretical point of view, where the relation between the foci number obtained from experiment and the *k*-value in the MK model is illustrated.

The effects on living cells (e.g. surviving fraction and the number of DSBs) depend not only on the type of radiation but also on the energy. For example, it is well known that low-energy electrons such as δ-rays have a larger impact than high-energy electrons [24]. However, the different effects by the same type of radiation with different energies have rarely been discussed in the literature. Although the RBE and radiation weighting factor (*W_R_*) are currently regarded as unity for electrons and photons, this energy dependence must be non-negligible for estimating the number of DSBs and the surviving fraction in the same cell line.

In this study, we take the parameter (*k*) in the MK model and establish a formulation for estimating the number of DSBs by deriving the rate equations for lesions after irradiation. For two types of cell lines, Chinese hamster ovary (CHO-K1) and human non-small cell lung cancer (H1299) cells, we attempt to evaluate the parameter (*k*) in the MK model by comparing the relative number of DSBs estimated by the *β* value in the LQ formula with the number of γ-H2AX foci. In parallel, we investigate the relationship between *y*_*D*_ and *k* estimated using the equation derived in the model. We sought to confirm that photon beams with lower energies induce more intensive damage in the cells. We also evaluate the validity of the equation for estimate the number of DSBs.

## MATERIALS AND METHODS

### Surviving fraction and *y*_*D*_ value in the MK model

The MK model was proposed by R.B. Hawkins in 1994 [12]. In this model, each cell nucleus is divided into several domains. The lesions arising from irradiation in a domain are called potentially lethal lesions (PLLs). A PLL can undergo one of four transformations: (i) it may be converted to a lethal and non-repairable lesion via a first-order process (rate constant for transformation is *a* [h^−1^]); (ii) it may be converted to a lethal and non-repairable lesion via a second-order process (rate constant for transformation is *b*_*d*_ [h^−2^]); (iii) it may be repaired via a first-order process (rate constant for transformation is *c* [h^−1^]); (iv) it may maintain the lesion state for a period of time *t*_*r*_ [h], after which time, if it is still present, it becomes lethal and non-repairable [12]. In the MK model, it is assumed that the PLLs are DSBs in DNA. Using the rate constants (*a*, *b*_*d*_ and *c*) for the transformations above, a rate equation of the number of PLLs per domain for a single-dose irradiation is expressed as:

(1) $$\displaystyle{{dP} \over {dt}}=- (a+c)P - 2b_d P^2 .$$

By considering the case of (*a* + *c*)*P* > >2*b*_*d*_*P*^2^,

(2) $$\displaystyle{{dP} \over {dt}} \cong - (a+c)P$$

and

(3) $$P=k_d ze^{ - (a+c)t} .$$

Here, *P* is the number of PLLs in a domain; *k*_*d*_ is the number of PLLs produced on average per dose [Gy^−1^] just after the irradiation; *z* is specific energy deposited in the domain [Gy]; *t* is time after the irradiation to the cell nucleus [h] and satisfies 0 < *t < t*_*r*_. The rate equation of the lethal lesions per domain (*L*) is expressed by:

(4) $$\displaystyle{{dL} \over {dt}}=aP+b_d P^2 .$$

By solving Eq. (4) after the substitution of Eq. (3) in the terms in the right hand side, we have

(5) $$L=Az+Bz^2 ,$$

where

(6) $$A=\displaystyle{a \over {(a+c)}}k_d+\displaystyle{{\rm c} \over {(a+c)}}k_d e^{ - (a+c)t_r } {\rm }$$

and

(7) $$B=\displaystyle{{b_d k_d ^2 } \over {2(a+c)}}[1 - e^{ - 2(a+c)t_r } ].$$

The average number of lethal lesions *L*_*n*_ per cell nucleus and the surviving fraction (*S*) for the single-dose irradiation can be described by using the expected value (with brackets), assuming the Poisson distribution of the number of lethal lesions in a cell nucleus, as follows:

(8) $$\eqalign{ L_n&=N\left\langle L \right\rangle=N\left( {A\left\langle z \right\rangle+B\left\langle {z^2 } \right\rangle } \right) \cr &=\left( {\alpha _0+\displaystyle{{\beta y_D } \over {\rho \pi r_d ^2 }}} \right)D+\beta D^2 \cr &=\alpha D+\beta D^2=- \ln S,} $$

where

(9) $$\alpha _0=NA,$$

(10) $$\beta=NB,$$

(11) $$k=Nk_d,$$

(12) $$\alpha=\alpha _0+\displaystyle{{\beta y_D } \over {\rho \pi r_d^2 }}$$

and *N* is the number of domains in a cell nucleus; $\left\langle L \right\rangle$ is the average number of lethal lesions per domain; *r*_*d*_ and ρ represent the radius of the domain (= 0.5 µm) and the density of the domain (= 1.0 g/cm^3^), respectively; *D* is the absorbed dose in Gy; *k* is the number of PLLs per cell nucleus; *α* and *β* represent LQ (Linear–Quadratic) parameters that are obtained by fitting to the cell surviving fraction data; the *y*_*D*_-value is the dose mean lineal energy (keV/μm). The *y*_*D*_-value is given by

(13) $$y=\displaystyle{\varepsilon \over {\left\langle l \right\rangle }}$$

and

(14) $$y_D=\displaystyle{{\int {y^2 f(y)dy} } \over {\int {yf(y)dy} }}=\displaystyle{{\int {yd(y)dy} } \over {\int {d(y)dy} }},$$

where ϵ is the energy deposited in a domain, <*l*> is the mean chord length expressed as two-thirds times the domain diameter (i.e. 2/3 × 1 µm water-equivalent), *y* is the lineal energy, *f*(*y*) is the probability density of the lineal energy, *d*(*y*) is the dose distribution of the lineal energy. Note that Eq. (8) for the surviving fraction in the MK model includes the dose mean lineal energy *y*_*D*_ in keV/μm.

### Principle for estimating the number of DSBs according to the MK model

Once it is assumed that the PLLs correspond to DSBs in DNA, the number of DSBs per cell nucleus (*k*) can be obtained by fitting Eq. (8) to the experimental surviving fraction. By substituting Eq. (7) into Eq. (10), *β* is derived as follows:

(15) $$\eqalign{& \beta=NB \cr &=\displaystyle{{Nb_d k_d^2 } \over {2\left( {a+c} \right)}}\left[ {1 - e^{ - 2\left( {a+c} \right)t_r } } \right] \cr &=\displaystyle{{bk^2 } \over {2\left( {a+c} \right)}}\left[ {1 - e^{ - 2\left( {a+c} \right)t_r } } \right],} $$

where *b* (= *b*_*d*_*/N*) is the rate constant for transformation to a lethal and non-repairable lesion via a second-order process [h^−1^]. If we assume that (*a* + *c*)*t*_*r*_ is > 3 following Hawkins [13], Eq. (15) can be approximated as:

(16) $$\beta \cong \displaystyle{{bk^2 } \over {2\left( {a+c} \right)}}.$$

Since (*a + c*) and *b* are regarded as specific parameters to a certain type of cells, the ratio of *β* to *k*^2^ would be constant as:

$$\displaystyle{\beta \over {k^2 }} \cong \displaystyle{b \over {2\left( {a+c} \right)}}=const.$$

Accordingly, if the parameters *β* and *k* are given for radiation with a certain energy (e.g. γ-ray), then the parameters *β'* and *k'* for radiation with some other energy can be related as:

(17) $$\displaystyle{{k^{\prime}} \over k}=\sqrt {\displaystyle{{\beta ^{\prime}} \over \beta }}.$$

From this relation, we can estimate the relative number of DSBs (*k'/k*) by the determination of *β* from the cell survival curves.

### Cell line, culture condition and irradiation

The CHO-K1 and H1299 cell lines were obtained from RIKEN Bio Resource Center in Japan and ATCC, respectively. Two cell lines were maintained in Dulbecco's modified Eagle's medium (DMEM, Sigma, St Louis, MO, USA) supplemented with 10% fetal bovine serum (FBS, Nichirei Biosciences Inc., Tokyo, Japan) at 37°C in a humidified 95% air, 5% CO_2_ incubator. Both cells were kept within vent cap flasks whose area was 25 cm^2^ (IWAKI, Tokyo, Japan), and were grown to semi-confluence. Each type of cell was irradiated with 200 kVp X-rays (Siemens, Concord, CA) at a dose rate of 1.25 Gy/min, or by 6 MV therapeutic X-rays (Mitsubishi Electric Co., Tokyo, Japan) at a dose rate of 2.5 Gy/min. In the case of kV photon irradiation, we measured the air dose rate at the surface of the cell culture using an ionization chamber (NE2571). In the case of MV photon irradiation, the dish containing the cells was placed on a water-equivalent phantom with a 50 mm-thickness at the side of the gantry head in order to compensate for the build-up effect. The absorbed dose in water (*D*) was determined according to the dose protocol of TRS 277 [25] (in air method) for 200 kVp X-rays and Japanese standard dosimetry 01 [26] for Linac-6 MV X-rays, respectively. Each irradiation was performed at room temperature.

### Clonogenic assay

The cells were grown to semi-confluence, irradiated, trypsinized and counted. 1 × 10^3^, 1 × 10^4^ or 1 × 10^5^ cells were reseeded in plastic dishes. The cells had been cultured in a CO_2_ incubator for 10–14 d. Then, the DMEM was replaced every 2 d. After the culture period, the cells were fixed with methanol and stained with 2% Giemsa solution (Kanto Chemical Co. Inc., Tokyo, Japan) to determine the number of colonies per dish. The colonies were counted and survival rates were calculated with the plating efficiency of the non-irradiated cells.

### Estimation of the DNA damage based on the MK model

To estimate the number of PLLs in a cell nucleus after irradiation of 1.0 Gy, we took the cell survival data from the literature. For CHO-K1 cells, Zellmer *et al*. (1998) have reported the surviving fraction with 200 kVp X-rays [27]. Using their data and our experimental results for irradiation with 6 MV X-rays for CHO-K1 cells, we deduced the parameters *α* and *β* by fitting the LQ formula to the data via the least squares method and confirmed the difference between the effects of two types of photon irradiation. The ratio of parameter (*k*) for 6 MV X-rays and for 200 kVp X-rays was then obtained. In the same manner, we obtained the ratio of *k* for H1299 cells using our survival data from the clonogenic assay.

### Immunofluorescence staining with γ-H2AX

Two types of cell lines, CHO-K1 and H1299 cells, were grown on the ϕ 35 mm glass cover slips covered with collagen, irradiated with 6 MV and 200 kVp X-rays at 1.0 Gy. At 30 min after the irradiation, the cells on the coverslips were fixed in a 4% paraformaldehyde solution with PBS for 10 min at room temperature and then rinsed three times with PBS. Next, the cells were permeabilized in ice-cold 0.2% Triton X-100 in PBS for 5 min, and blocked with a solution of 1% BSA-containing PBS for 30 min. After that, a primary antibody γ-H2AX (diluted by a solution of 1% BSA-containing PBS) was fed into the glass coverslips, and kept overnight at 4°C. The next day, the primary antibody was removed and rinsed three times with a solution of 1% BSA-containing PBS. Then Alexa Fluor 594-conjugated goat–anti–rabbit (Molecular Probes, Invitrogen, Japan), diluted by a solution of 1% BSA-containing PBS, was put onto coverslips and left for 2 h, then rinsed one time with 1 µg/ml DAPI (4′,6-diamidino-2-phenylindole phenylindole)-containing methanol. The cells on the coverslips were stained with 1 µg/ml DAPI-containing methanol for 15 min in the incubator, and rinsed one time with methanol.

The γ-H2AX foci in the cell nuclei were observed using a High Standard all-in-one fluorescent microscope (model BZ-9000; Keyence, Osaka, Japan). The images of γ-H2AX foci were taken using the Z-stack function, and reconstructed by quick full focus with 3D in the cell nucleus. The number of the γ-H2AX foci was counted using VH-H1A5 software (Keyence, Tokyo, Japan), and presumably corresponds to the number of DSBs. This procedure was conducted for each condition (control and 1.0 Gy in each cell line, for more than 80 cells of CHO-K1 or H1299). In the measurement of the foci, binarized processing was adopted, setting the threshold level of fluorescence intensity corresponding to the visible level.

### Comparison between *k* ratio in the MK model and ratio of γ-H2AX foci number

The γ-H2AX foci number per cell nucleus per Gy obtained in the previous subsection was defined as experimental *k*, written as *k*_*exp*_ in this paper. Using the results of *k*_*exp*_ for both cell lines, the ratio of *k*_*exp*_ for 6 MV X-rays and for 200 kVp X-rays was calculated. The ratio of *k*_*exp*_ was then compared with the ratio of *k* estimated based on the MK model (Eq. (17)). From the result of ratios, we evaluated the parameter (*k*) in the MK model.

### Analysis of the relationship between the *y*_*D*_-value and the number of DSBs

In order to confirm the dependence of cell survival on photon energy, we collected the cell survival data previously reported in the literature (listed in Table 1) for CHO-K1 cells exposed to 100 kVp X-rays [28], ^60^Co γ-rays [29–32] and ^137^Cs γ-rays [27, 33–39], and for H1299 cells exposed to ^60^Co γ-rays [40] and ^137^Cs γ-rays [41–43]. Here, the parameters *α* and *β* were determined in the same manner as mentioned above. The *k*_*exp*_ after irradiation with 200 kVp X-rays was adopted as the standard radiation; then by using Eq. (17), we estimated each *k* exposed to each photon type (6 MV X-rays, ^137^Cs γ-rays, 200 kVp X-rays and 100 kVp X-rays). In addition, the RBE value was calculated at the 37% survival level, which is related with the number of DSBs.

**Table 1.** A list of references for survival data

Cell line	Radiation Type	Reference [27–43]
CHO-K1	^60^Co γ-rays	Mothersill C *et al.*, 1986 [29]
		Czub J *et al.*, 2008 [30]
		Murakami D *et al.*, 2004 [31]
		Thacker J *et al.*, 1985 [32]
	Linac-6 MV X-rays	present (clonogenic assay)
	^137^Cs γ-rays	Schneiderman MH *et al.*, 2001 [33]
		Schwartz JL *et al.*, 2000 [35]
		Van Putten JWG *et al.*, 2001 [34]
		Woudstra EC *et al.*, 1999 [36]
		Stevens CW *et al.*, 1999 [37]
		Murray D *et al.*, 2000 [38]
		Britten RA *et al.*, 1997 [39]
	200 kVp X-rays	Zellmer, DL *et al.*, 1998 [27]
	100 kVp X-rays	Wedemeyer N *et al.*, 2001 [28]
H1299	^60^Co γ-rays	Iwasa T *et al.*, 2009 [40]
	Linac-6 MV X-rays	present (clonogenic assay)
	^137^Cs γ-rays	Liu S-K *et al.*, 2008 [41]
		Chen M-F *et al.*, 2006 [42]
		Shin S-H *et al.*, 2007 [43]
	200 kVp X-rays	present (clonogenic assay)

## RESULTS

### Parameters in the MK model using the surviving fraction

**Fig. 1.**
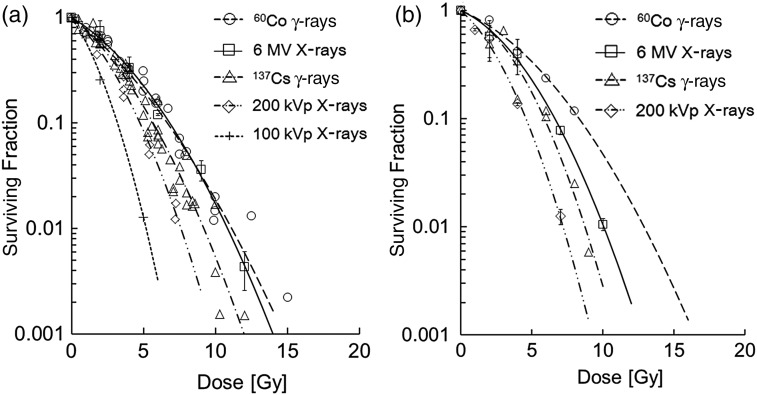
Surviving fractions as a function of dose after the irradiation of several types of photon beams: (**a**) for CHO-K1 cells, and (**b**) for H1299 cells. The lines fitted to the symbol plots were determined based on the MK model. The experimental data were taken from the literature [27–43] listed in Table 1.

Figure 1a and b shows the cell surviving fractions for CHO-K1 cells and for H1299 cells, respectively, after irradiation with a variety of photon beams. The survival curves in the figures fit well to the experimental data listed in Table 1. The curves exhibit a general tendency that the surviving fraction of cells exposed to lower energy photons was lower than that of cells exposed to higher energy photons, even at the same absorbed dose, i.e., the surviving fraction for 200 kVp X-rays was less than that for 6 MV X-rays. Table 2 presents a list of the parameters in the MK model, which was obtained by fitting Eq. (8) to the surviving fraction. Here, the *α* and *β* values were determined as two independent parameters. It should be noted that the *β* value decreases with photon energy.

**Table 2.** MK parameters (*α, β, α*_*0*_*, y*_*D*_*, k*), experimental *k* (obtained by counting the number of γ-H2AX foci) and RBE_37_ for CHO-K1 and H1299 cell lines

Cell line	Radiation Type	*a*	*α*_*0*_	*β*	*y*_*D*_ [keV/ μm]	*k*	*k*_*exp*_	RBE_37_
CHO-K1	^60^Co γ-rays	0.246 ± 0.023	0.238 ± 0.024	0.0152 ± 0.0023	2.34	30.4 ± 9.3	30^a^	0.757
	Linac-6 MV X-rays	0.190 ± 0.034	0.179 ± 0.035	0.0218 ± 0.0033	2.36	36.5 ± 11.2	39.4 ± 13.1	0.689
	^137^Cs γ-rays	0.255 ± 0.029	0.239 ± 0.032	0.0270 ± 0.0036	2.90	40.6 ± 12.3		0.855
	200 kVp X-rays	0.284 ± 0.051	0.245 ± 0.070	0.0425 ± 0.0083	4.51	50.9 ± 14.1	50.9 ± 14.1	1.000
	100 kVp X-rays	0.456 ± 0.082	0.378 ± 0.135	0.0833 ± 0.0179	4.70	71.3 ± 22.7		1.522
H1299	^60^Co γ-rays	0.130 ± 0.033	0.121 ± 0.036	0.0176 ± 0.0048	2.34	33.6 ± 9.2		0.539
	Linac-6 MV X-rays	0.137 ± 0.036	0.121 ± 0.038	0.0319 ± 0.0041	2.36	45.3 ± 10.8	37.9 ± 13.7	0.659
	^137^Cs γ-rays	0.113 ± 0.074	0.085 ± 0.083	0.0474 ± 0.0103	2.90	55.2 ± 14.2		0.713
	200 kVp X-rays	0.277 ± 0.048	0.231 ± 0.066	0.0526 ± 0.0078	4.51	58.1 ± 12.5	58.1 ± 12.5	1.000

^a^Indicates the nominal number of DSBs recommended in the reference [13, 44]. The error of *k* value and *α*_0_ value were estimated by propagation of error from *β* value and *α* value.

By using the *β* values with 200 kVp X-rays and 6 MV X-rays in Table 2, we estimated the ratio of DSBs using Eq. (17). In CHO-K1 cells, the ratio of *k* for irradiation by 6 MV X-rays to that for irradiation by 200 kVp X-rays was estimated to be 0.716 ± 0.088, while in H1299 cells it was 0.779 ± 0.076. These ratios suggest that the number of DSBs resulting from 200 kVp X-rays is larger than that from 6 MV X-rays, independent of cell type.

**Fig. 2.**
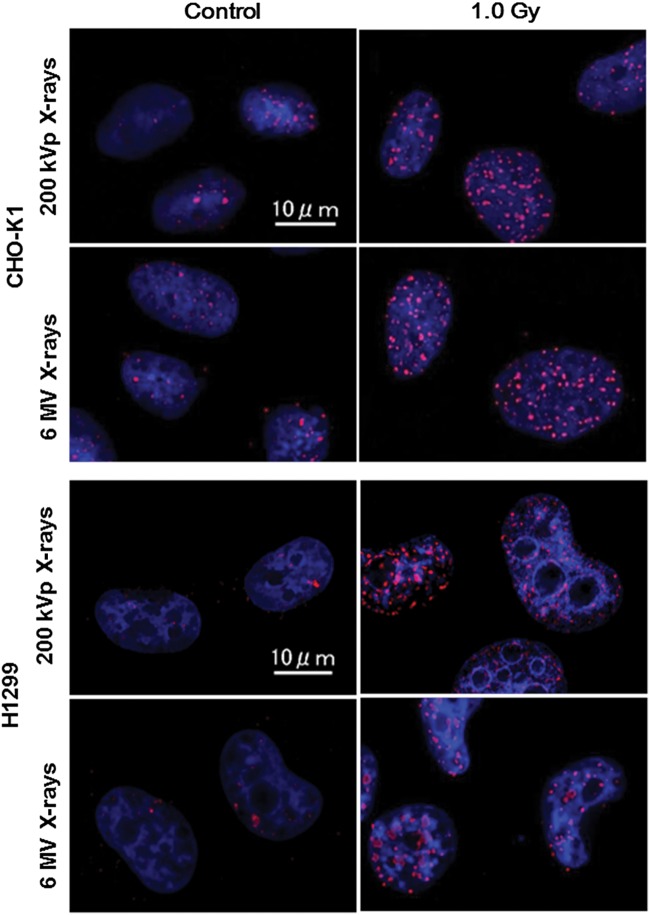
Images in the γ-H2AX antibody fluorescent observation for CHO-K1 and H1299 cells. The blue area represents a cell nucleus, and red points are the γ-H2AX foci indicating DNA double-strand breaks (DSBs).

**Fig. 3.**
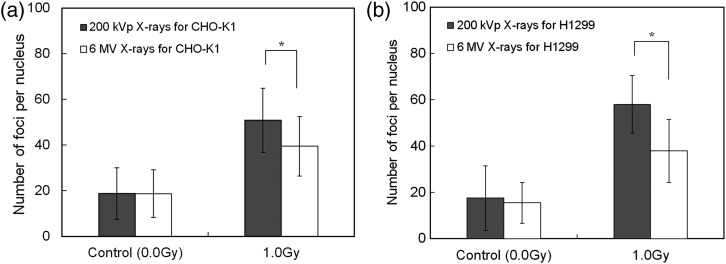
Number of DSBs obtained by counting the γ-H2AX foci: (**a**) for CHO-K1, and (**b**) for H1299 cells. The asterisk represents a *P* < 0.05 significant difference.

The γ-H2AX foci observed in this study are shown in Fig. 2, where the blue area represents a cell nucleus stained with DAPI, and each red point represents a DSB. The number of γ-H2AX foci (*k*_*exp*_) was counted using VH-H1A5 software (Keyence, Tokyo, Japan), and is regarded as the number of DSBs. The γ-H2AX foci numbers per cell nucleus are shown in Fig. 3a for CHO-K1 cells and in Fig. 3b for H1299 cells, respectively. The average numbers of foci per nucleus before irradiation (background) for CHO-K1 and H1299 cells were 18.7 ± 7.69 and 16.5 ± 8.24, respectively. These background numbers of foci were at a comparable level for 6 MV and 200 kVp X-rays. After irradiation of 1.0 Gy, *k*_*exp*_ for CHO-K1 cells irradiated with 200 kVp and 6 MV X-rays was 50.9 ± 14.1 and 39.4 ± 13.1, respectively; *k*_*exp*_ for H1299 cells similarly irradiated was 58.1 ± 12.5 and 37.9 ± 13.7, respectively. The ratio of *k*_*exp*_ for 6 MV X-rays to *k*_*exp*_ for 200 kVp X-rays with CHO-K1 cells was 0.774 ± 0.335 and for H1299 cells it was 0.652 ± 0.274. The DSB ratio from γ-H2AX observation (*k*_*exp*_ ratio) closes in on the value of the ratio of *k* estimated by the MK model, convincing us that the parameter (*k*) in the MK model corresponds to the number of DSBs per cell nucleus.

### Comparison of the parameters with other data, and evaluation of *y*_*D*_

Based on Eq. (17) and the results of γ-H2AX foci observation (*k*_*exp*_) for 200 kVp X-ray irradiation as the standard radiation (i.e. 50.9 ± 14.1 per nucleus per Gy for CHO-K1 cells in Fig. 3a, and 58.1 ± 12.5 per nucleus per Gy for H1299 cells in Fig. 3b), we estimated the numbers of DSBs per cell nucleus (*k*) for the other cases with 100 kVp X-rays, ^137^Cs γ-rays, 6 MV X-rays and ^60^Co γ-rays for CHO-K1 cells, and 6MV X-rays and ^60^Co γ-rays for H1299 cells, to be as shown in Table 2. In general, the *k* estimated by the MK model fits well the number of γ-H2AX foci (*k*_*exp*_). The nominal number of DSBs for Chinese hamster ovary cell lines was taken from the reference [12, 44], and we adopted 30 as the expected *k*_*exp*_ with ^60^Co γ-rays for CHO-K1 cells. As is shown in Table 2, the number of DSBs (*k*) increases with the dose mean lineal energy *y*_*D*_ [keV/μm], which suggests that *k* depends on the energy of photon beams. Table 2 also shows that the RBE_37_ is generally elevated along with the *k* value.

## DISCUSSION

### Parameter *k* in the MK model and the number of DSBs *k*_*exp*_

We have paid attention primarily to the parameter *k* in the MK model in this study. Since the PLLs are assumed to be DSBs in the MK model, *k* can be regarded as the number of DSBs per cell nucleus per Gy. As shown in Table 2, the *k* estimated in the MK model agreed well with the number of γ-H2AX foci (*k*_*exp*_), supporting this assumption. The *k*-value was estimated from *β* in this study. According to the methodology of the MK model, we can deduce a *k*-value from *α*_0_ as well. However, it is expected that the *k*-value from *α*_0_ will have an uncertainty larger than that from *β* (as shown in Table 2) because *α*_0_ is deduced from *α* and *β*, while *β* is directly determined by a curve fitting to survival data. Therefore, it might be reasonable to estimate the *k*-value from *β* via Eq. (17).

It is known that the stopping power of electrons has a peak in the low-energy region around 100 eV in liquid water [45], which brings about the peak energy deposition just before the electrons stop. In fact, there are many ionization and excitation events in the vicinity of the track end [46]. This concentration of energy deposition around the track end should have a larger influence on the probability of inducing lesions in a cell nucleus than the other parts of the track. The ratio of the energy depositions around the track end to the other track parts is larger for a short track with a low-energy electron than that for a long track with a high-energy electron. Therefore, even at the same absorbed dose, the damage probability caused by a low-energy electron produced through low-energy photon irradiation is larger than that caused by a high-energy electron produced through high-energy photon irradiation. Further to this, the estimated numbers of DSBs (*k*) in Table 2 evidently reflect the degree of damage inflicted by secondary electrons produced after several types of photon irradiation.

### Evaluation of the effect by photon beams depending on their energies

**Fig. 4.**
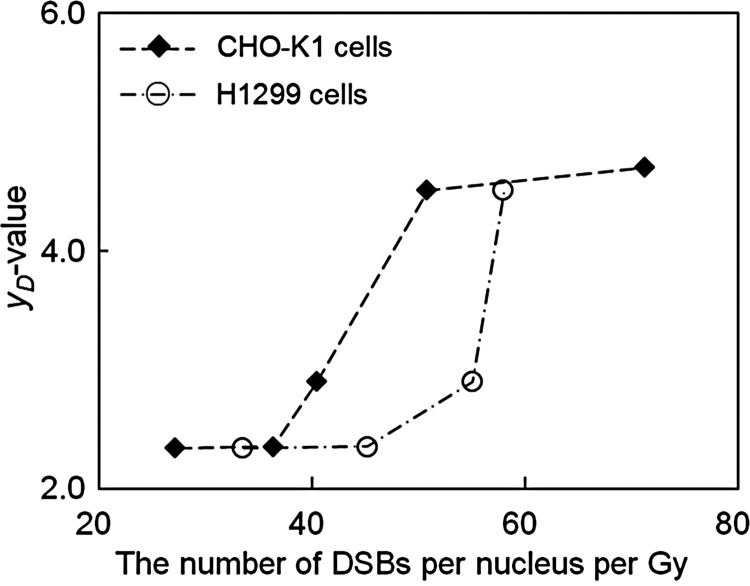
Relationship between the dose mean lineal energy *y*_*D*_ (keV/μm) and the number of DSBs (per cell nucleus per Gy): closed diamonds for CHO-K1 cells, and open circles for H1299 cells. The numbers of DSBs were estimated using Eq. (17).

We also investigated the relationships between the dose mean lineal energy *y*_*D*_ [keV/μm], the RBE_37_ value and the number of DSBs per cell nucleus per Gy (*k*). As shown in Fig. 4, the number of DSBs per nucleus per Gy increases monotonically as the *y*_*D*_ value increases. This result may have arisen from the characteristic that *y*_*D*_ depends on the photon energy, as indicated in Table 2. The number of DSBs detected by the γ-H2AX antibody (*k*_*exp*_) shows a clear trend, i.e. that lower energy X-rays induce DSBs more than higher energy X-rays. This is attributable to the energy deposition pattern along the secondary-electron track, which concentrates on the track end, as mentioned in the previous subsection. Since *y*_*D*_ represents the energy deposition at every site, including the end part, lower energy electrons arising from low-energy photon irradiations will increase the *y*_*D*_ value in a cell nucleus. As we can see in Table 2, the magnitude relationship between the number of DSBs with ^60^Co γ-rays and that with 6-MV X-rays is opposite to the relationship between the mean energies of both photon beams (the mean energy of ^60^Co γ-rays is 1.25 MeV, and the mean energy of continuous 6-MV X-rays is roughly estimated to be 1.70 MeV). This result must be explainable on the energy spectra of electrons created by two types of photon beams. We supposed that *y*_*D*_ reflects the biological effect along with the microscopic electron processes, which can be verified from the viewpoint of the dose mean lineal energy, *y*_*D*_ in keV/μm, not from the mean energy of electrons. Table 2 shows that the value of *k* (supposedly the number of DSBs) lies in the same order of the value of *y*_*D*_, supporting this presumption.

**Fig. 5.**
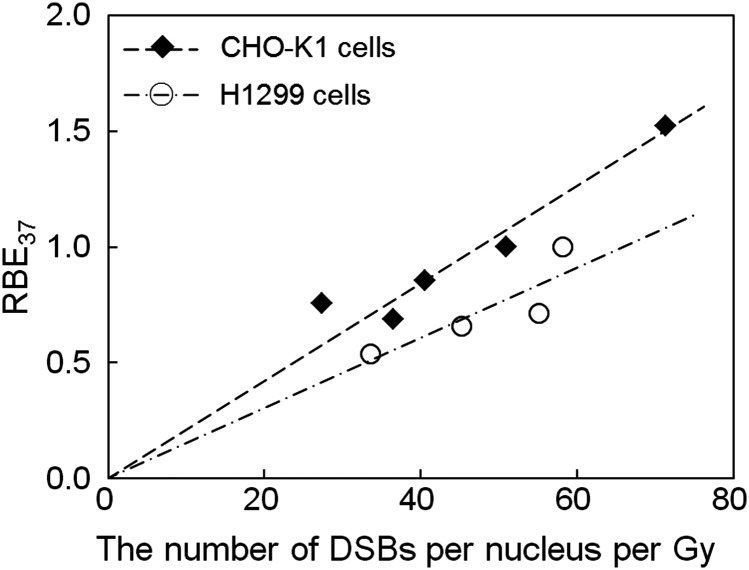
Relationship between RBE_37_ and the number of DSBs (*k*). The number of DSBs was estimated using Eq. (17) while the RBE_37_ value was deduced from the absorbed dose at 37% survival level using Eq. (8).

In addition to *y*_*D*_, by replacing the surviving fraction with RBE_37_, the relationship between RBE_37_ and *k* [estimated number of DSBs per nucleus per Gy based on Eq. (17)] is given in Fig. 5 as well. The RBE_37_ value is likely to be proportional to the number of DSBs. Alternatively the RBE value can be obtained by calculating the relative number of DSBs (when we see the number of DSBs as an endpoint of cell damage) [47]. Since the parameter *k* is proportional to the number of foci, the ratio of *k* has to be proportional to the RBE. The proportional relation between *k* and RBE_37_ in Fig. 5 suggests the possibility of evaluating the effects on cells and DSB production from the viewpoint of biodosimetry [48, 49].

## CONCLUSION

In this study, we evaluated the relationship between *k* (as the number of DSBs) and *y*_*D*_, and also *k* and RBE_37_. It was shown that *k* deduced using the MK model agrees well with the number of DSBs per cell nucleus that is observed by immunofluorescent staining using γ-H2AX antibody. Further, the surviving fraction at the same dose decreases as the *y*_*D*_ value increases, while both the DSB number and the RBE_37_ value increase with *y*_*D*_. These results suggest that the effects of photons on cell survival and the DNA damage depend on the photon energy in terms of both microdosimetry and biodosimetry. The dependence on the photon energy is explainable from the energy deposition along the track of electrons (created by the photon) passing through the cell nucleus. The present study suggests that the RBE value and the radiation weighting factor (*W_R_*) for electrons and photons should not always be treated as unity. It was also demonstrated that a quantitative estimation of DNA damage can be made through the perspective of energy deposition along the electron track, which is promising for evaluating the damage effects on cells exposed to ionizing radiation.

## FUNDING

Funding to pay the Open Access publication charges for this article was provided by H. Date (Hokkaido University).
